# Timing of antibiotics, volume and vasoactive infusions in children with sepsis: it is all in the timing

**DOI:** 10.1186/s13054-015-1122-3

**Published:** 2015-11-21

**Authors:** Samiran Ray, Mark J. Peters

**Affiliations:** Respiratory, Critical Care and Anaesthesia Section, UCL Institute of Child Health, 30 Guildford Street, London, WC1N 1EH UK; Paediatric and Neonatal Intensive Care Unit, Great Ormond Street Hospital, London, WC1N 3JH UK

We share the view of van Paridon et al. [1] that robust data on fluid bolus therapy for sepsis resuscitation are needed, and looked with interest to the Alberta Sepsis Network’s recent report to add to our understanding. We note that the authors chose to exclude children who were expected to die <24 hours from presentation. We would be grateful to know how many cases were excluded on this basis. The majority of the hazard from sepsis lies in this period. We recently defined this risk in a similar cohort of children; 55 % of all sepsis deaths (78 % of these being previously healthy children) occurred within the first 24 hours [2].

Resuscitation interventions such as fluid and vasoactive drugs are guided by changes in physiology. Inasmuch as physiological variables are associated with mortality, these interventions are also likely to have an immediate effect on mortality. The authors suggest potential explanations for the 1-year mortality effect seen with aggressive fluid administration: while all are not only plausible but likely, the effect will only be seen if the acute phase is overcome. By excluding children dying early, the authors also reduce the potential to propensity match children receiving similar levels of interventions. Pediatric Risk of Mortality (PRISM) scores control for severity of illness, but information is lost given that the PRISM score utilises categories of haemodynamic variables rather than age-standardised continuous variables [3]. We wonder whether the modest effect size difference would persist with these alterations to the methodology.

## Abbreviations

PRISM: Pediatric Risk of Mortality
