# Supplementary material for: Timing of antibiotics, volume and vasoactive infusions in children with sepsis: it is all in the timing
Source: Crit Care. 2015 Nov 21;19:394. doi: 10.1186/s13054-015-1122-3 (PMC4654822; doi:10.1186/s13054-015-1122-3)
Supplement: Related texts

# Reply: Authors’ response

**Authors:** Bregje M van Paridon, Cathy Sheppard, Gonzalo Garcia Guerra, Ari R Joffe

We thank Ray and Peters for their interest in our study. They point out that we excluded children who were expected to die within 24 hours from presentation, and that these are the children who may be most likely to benefit from aggressive fluid resuscitation. They hypothesise that aggressive fluid administration may be non-beneficial only once the ‘acute phase’ is overcome.

We agree this is a limitation of our study. In our cohort of children screened for sepsis in the paediatric ICU, one child was excluded because survival for >24 hours was not expected and one child was excluded because palliative care had been decided upon. Although we do not have exact numbers, the number of children with sepsis presenting to our Children’s Hospital emergency department or referral area and dying before arriving in the paediatric ICU is extremely small. In addition, although only one patient in our cohort died in the paediatric ICU, six children required ECLS (extracorporeal life-support), an indicator of the severity of sepsis [4]. Thus, we believe the number of patients excluded from our study who died within 24 hours after sepsis presentation is very small. In addition, we note that recent editorials continue to point out the variability in fluid bolus therapy practice, the limited data on effectiveness of fluid bolus therapy and that the ideal resuscitation target is a knowledge gap in sepsis [5–7]. Although we do not recommend a change in practice at this time, we believe that further study of fluid bolus therapy and alternative strategies for resuscitation in sepsis are warranted.
